# Understanding and Addressing Challenges With Electronic Health Record Use in Gynecological Oncology: Cross-Sectional Survey of Multidisciplinary Professionals in the United Kingdom and Co-Design of an Integrated Informatics Platform to Support Clinical Decision-Making

**DOI:** 10.2196/58657

**Published:** 2025-09-10

**Authors:** Laura Tookman, Rachael Lear, Yusuf S Abdullahi, Amit Samani, Phoebe Averill, Ashton Hunt, Dimitri Papadimitriou, Baleseng Elizabeth Nkolobe, Sadaf Ghaem-Maghami, Ben Glampson, Iain A McNeish, Erik K Mayer

**Affiliations:** 1 iCARE Secure Data Environment & Digital Collaboration Space, NIHR Imperial Biomedical Research Centre, London, United Kingdom; 2 Imperial College Healthcare NHS Trust, Hammersmith Hospital, Du Cane Road, London, W12 0HS, United Kingdom, 44 02033131151; 3 Department of Surgery and Cancer, Faculty of Medicine, Imperial College London, London, United Kingdom; 4 Robin Hood Lane Health Centre, Central Sutton Primary Care Network, United Kingdom; 5 NIHR North West London Patient Safety Research Collaboration, Institute of Global Health Innovation, Imperial College London, United Kingdom

**Keywords:** digital health, electronic health records, clinical decision support, artificial intelligence, co-design, coproduction, ovarian cancer, gynecological oncology

## Abstract

**Background:**

Electronic health records (EHRs) are a cornerstone of modern health care delivery, but their current configuration often fragments information across systems, impeding timely and effective clinical decision-making. In gynecological oncology, where care involves complex, multidisciplinary coordination, these limitations can significantly impact the quality and efficiency of patient management. Few studies have examined how EHR systems support clinical decision-making from the perspective of end users. This study aimed to explore multiprofessional experiences of EHR use in gynecological oncology and to develop a co-designed informatics platform to improve decision-making for ovarian cancer care.

**Objective:**

This study aims to evaluate the perspectives of health care professionals on retrieving routine clinical data from EHRs in the management of ovarian cancer and to design an integrated informatics platform that supports clinical decision-making.

**Methods:**

We conducted a national cross-sectional survey of 92 UK-based professionals working in gynecological oncology, including oncologists, nurses, radiologists, and other specialists in ovarian cancer. The web-based questionnaire, combining quantitative and free-text responses, assessed their experiences with EHR use, focusing on information retrieval, usability challenges, perceived risks, and benefits. In parallel, a human-centered design approach involving health care professionals, data engineers, and informatics experts codeveloped a digital informatics platform that integrates structured and unstructured data from multiple clinical systems into a unified patient summary view for clinical decision-making. Natural language processing was applied to extract genomic and surgical information from free-text records, with data pipelines validated by clinicians against original clinical system sources.

**Results:**

Among 92 respondents, 84 out of 91 (92%) routinely accessed multiple EHR systems, with 26 out of 91 (29%) using 5 or more. Notably, 16 out of 92 respondents (17%) reported spending more than 50% of their clinical time searching for patient information. Key challenges included lack of interoperability (35/141 reported challenges, 24.8%), difficulty locating critical data such as genetic results (57/85 respondents, 67%), and poor organization of information. Only 10 out of 92 professionals (11%) strongly agreed that their systems provided well-organized data for clinical use. While ease of access to patient data was a key benefit, 54 out of 90 respondents (60%) reported lacking access to comprehensive patient summaries. To address these issues, our co-designed informatics platform consolidates disparate patients’ data from different EHR systems into a single visual display to support clinical decision-making and audit.

**Conclusions:**

Current EHR systems are suboptimal for supporting complex gynecological oncology care. Our findings highlight the urgent need for integrated, user-centered clinical decision tools. Fragmentation and lack of interoperability hinder information retrieval and may compromise patient care. Our co-designed ovarian cancer informatics platform is a potential real-world solution to improve data visibility, clinical efficiency, and ultimately the quality of ovarian cancer care.

## Introduction

Electronic health records (EHRs) have been widely adopted in modern health care systems and have evolved over time to develop functionality including clinical decision support systems (CDSSs), computerized provider order entry systems, and health information exchange between organizations—offering to improve the quality, safety, and efficiency of services [1]. By providing access to patient information in a timely manner, EHRs should allow health care professionals to spend more time with patients and reduce duplication of work; however, they are also known to increase administrative burden for clinical teams and even contribute to burnout [2-5].

As the digital transformation of health care has continued, much has been learned about the factors that promote successful EHR implementation, and the usability experiences of health care professionals have been identified as pivotal to EHR systems realizing their full potential [2,6]. Despite the vast amount of evidence published on EHRs and extensive debate among health care professionals in the nonempirical literature, relatively few studies have explored the experiences of frontline clinical teams as the primary end users of EHRs [2,7]. Studies focusing on the perspectives of health care professionals consistently report the same perceived benefits, including ease of access to real-time patient information and the possibility to view the longitudinal patient record [6,8]. However, dominating these study findings are the EHR usability concerns of end users—specifically, a lack of integration into clinical workflows, large volumes of poorly organized and unstructured information (“note bloat”), difficulties locating essential data for clinical decision-making, limited interoperability with other clinical systems and organizations, and alert fatigue [6,8,9]. Many of these challenges arise because of variation in EHR systems, clinical protocols, and documentation practices across health care organizations, leading to a significant amount of “noise” in the EHR [10]. A future generation of EHR systems is likely to embed clinical decision support tools driven by artificial intelligence and machine learning to reconcile fragmented data across by extracting clinically pertinent information and transforming it into coded data to create a higher-quality, more usable record that provides health care professionals with the right information, in the right place, at the right time [4,11].

The benefits and challenges of EHRs are widely recognized across clinical specialties, but the effects of EHR use, both positive and negative, may be particularly significant in oncology [3,6-9,12]. Cancer care is complex and high-risk, often characterized by multiple interventions delivered by diverse professionals, in a variety of health care settings, and over extended periods of time [13]. This is exemplified in gynecological oncology, where the patient pathway moves between the surgical and oncology teams (often at multiple time points), and management involves multiple health care specialties including geneticists, which is vital for clinical decision-making.

EHRs are designed to serve all clinical specialties and are subject to broad regulatory and institutional requirements with limited EHR specialization for end users delivering cancer care [14]. There is some limited evidence to support the application of EHR-based CDSSs that are specific to cancer care; for example, studies evaluating the use of oncology-focused computerized provider order entry systems have demonstrated positive impacts on prescriber errors, medication safety events, and clinical workflows [15]. However, despite low adoption rates for cancer-specific CDSSs, few studies have explored multiprofessionals’ perspectives and experiences on using EHR systems [16]. This study explored multiprofessional perspectives on using EHRs in gynecological oncology and co-designed an informatics platform to bring together key information for clinical decision-making in ovarian cancer.

## Methods

### Study Design

The study comprised 2 components: a national cross-sectional survey using a web-based questionnaire and co-design of an informatics platform. The survey was designed to assess the experiences of EHR use by multidisciplinary professionals specializing in gynecological malignancies. In parallel, data engineers and informatics experts worked together with health care professionals to coproduce a digital informatics platform that brings together data for patients with ovarian cancer held across multiple EHR systems into a single location.

### Survey Questionnaire

The bespoke 17-item questionnaire was designed around three main themes: (1) retrieving information from EHRs to manage individual patients with gynecological cancers; (2) retrieving information from EHRs for service evaluation, audit, and research in gynecological cancer; and (3) the perceived challenges, benefits, and risks of EHRs. Themes 1 and 3 are reported here. Answers were recorded as multiple-choice options (tick box), Likert-type variables (indicating strongly agree, somewhat agree, etc), or narrative responses (text box) (Multimedia Appendix 1). The questionnaire was co-designed by multiprofessional experts in ovarian cancer (LT and AS) and a mixed methods researcher (RL), and was piloted with diverse health care professionals who specialize in gynecological oncology: 2 nurses, an oncologist, and a radiologist to ensure meaningful content and readability across a range of professional groups. Based on feedback, minor changes were made to the wording of some questions. See Multimedia Appendix 1 for the final questionnaire.

### Study Population

The target population was secondary care multidisciplinary professionals working in gynecological oncology including medical and clinical gynecological oncologists, nurses, allied health professionals, pathologists, radiologists, and multidisciplinary team (MDT) coordinators. There were no exclusions for age, sex, work location, or professional background.

### Data Collection

The questionnaire was administered on the web using Microsoft Forms; a pragmatic sampling strategy was selected to reach diverse professional groups. A web link was circulated via social media (iCARE Twitter) and the mailing lists of the British Gynaecological Cancer Society, Institute of Cancer Research, Association of Cancer Physicians, The British Association of Gynaecological Pathologists, UK Oncology Nursing Society, and the British Society of Urogenital Radiology. *S*urvey respondents were multidisciplinary professionals who work in the field of gynecological cancer in the United Kingdom, including medical oncologists, clinical oncologists, gynecological oncologists, clinical nurse specialists, dietitians, physiotherapists, pathologists, radiologists, and MDT coordinators. No personal or identifiable data were collected. Upon clicking the link, the participant was prompted to answer the survey questions. An additional statement at the end of the questionnaire reminds respondents that clicking the “Submit” button will constitute consent to participate. The survey was open for completion between July 1, 2023, and July 30, 2023; a reminder was sent 1 week prior to the survey closing.

### Analysis

Quantitative data were analyzed using descriptive statistics; data are presented as counts (n) and proportions (%). Free-text data were analyzed using content analysis [17-19]. The units of analysis were keywords and sentence fragments. Within the free-text responses, manifest content analysis was applied, which focuses on describing what is observable in a text, permits counting the frequency of specific codes or content (summative content analysis), and produces descriptive categories rather than themes [17,20,21]. One researcher with clinical and qualitative expertise (RL) gained familiarity with free-text responses through iterative review, making inductive notes on patterns observed within these data. Notes were used to shape a preliminary coding scheme, using the constant comparative technique [22]. The preliminary coding scheme was then shared and refined among the wider authorship team, including experts in gynecological oncology, to ensure validity and clarity in the operationalization of codes. To permit assessment of interrater reliability, 2 researchers with qualitative expertise (RL and PA) then systematically applied the coding scheme to all free-text survey responses. Coding was conducted independently, such that researchers were blind to the codes applied by the other coder. Coding disagreements were discussed amongst the 2 coders to reach consensus; it was planned that a member of the wider authorship team would act as an independent arbitrator where agreement could not be reached between the 2 coders, but this was not required in practice. Interrater reliability was calculated using Cohen κ. Member checking could not be performed as the questionnaire was anonymous [23].

### Co-Design of an Informatics Platform to Support Clinical Decision-Making in Ovarian Cancer

Human-centered design emphasizes the problems experienced by people and how design can respond to meet user needs [24]. A widely recognized visualization depicting the human-centered design process is the British Design Council’s Double Diamond Model, which focuses initially on understanding and defining the problem and then builds on the insights and experiences of users to co-design solutions [25]. A digital informatics platform was developed by bringing together key information from different clinical systems to be displayed in a single location for multidisciplinary gynecological oncology teams making decisions about individual patients with ovarian cancer. The informatics platform was co-designed by Imperial College Healthcare National Health Service (NHS) Trust’s (ICHT’s) gynecological oncology team in collaboration with data engineers and informatics experts from the Imperial Clinical Analytics Research and Evaluation (iCARE) team. iCARE is the trust’s Secure Data Environment (SDE)—a cloud-based, big data analytics platform that enables CDSSs and other data-driven interventions to be developed and tested in a secure way. Platform co-design and national survey administration were undertaken concurrently.

Stakeholders included medical oncologists (n=3), gynecological oncology surgeons (n=1), chemotherapy nurse specialists (n=1), and data engineers and informatics experts from the iCARE team (n=3). Over a 6-month period, virtual (Microsoft Teams) meetings (30 minutes to 1 hour) enabled close collaboration between the clinical and informatics teams. At the start of the project, these meetings took place weekly. As the project evolved, these moved to monthly depending on the requirements; overall, 12 meetings took place over the course of the project. The agenda initially focused on the clinical team’s data requirements and the location of relevant data in different clinical systems and, later, designing the visual displays for the platform. Draft sketches were developed by the clinical team and based on the clinical team’s requirements; the relevant structured and unstructured data were curated by data engineers within the iCARE SDE, and data feeds from the following clinical systems were established: (1) Somerset Cancer Register—diagnostic data (including the International Federation of Gynecology and Obstetrics stage and histology) and demographics. (2) Cerner Millenium—demographic data, surgical data, genomic reports, outpatient pharmacy encounters, and visit data. (3) ARIA Oncology Information System (Siemens and Varian)—systemic anticancer therapy data and European Cooperative Oncology Group performance status. (4) National Health Service (NHS) spine—date of death. (5) North West London Pathology—Sunquest Laboratory Information Management Systems—hematology, biochemistry, tumor markers, and histology.

Natural language processing (NLP) algorithms were developed to transform free-text documentation in operation notes and genomic results into structured outputs for digital platforms; algorithm development required close collaboration between the clinicians and the informatics team during an iterative process of coding, clinically verifying, and refining the models. Visualization of the data points was optimized by the data engineers. All iterations of the platform were discussed until consensus was achieved. The findings from the national survey confirmed the data points that are challenging to access within electronic records but need to be visualized for patient care, and these were assimilated into the final design. The final design included individual patient summary views and a cohort view displaying aggregated data for clinical audit. A data engineer created the user interface for the platform in Qlik Sense, and the direct care clinical team validated the accuracy of the data feeds by checking the information displayed in the platform against the source data in front-end clinical systems.

### Ethical Considerations

This project was approved by the Imperial College Research Ethics Committee (ID 6608217) and the National Institute for Health and Care Research Imperial Biomedical Research Centre Data Access and Prioritisation Committee (Database: iCARE—Research Data Environment; research ethics committee reference: 21/SW/0120) and was registered with the Imperial College Healthcare NHS Trust audit team (registration ID 891). We hold patient privacy at the center of all we do, and we manage, through robust review and procedures, data access, deidentification, and ethics with the utmost care, consideration, and respect. Our SDE is built in line with the Health Data Research UK safes and enables secure approved access to data within the NHS for researchers. This allows controlled access to deidentified data (in secure locked-down accounts), and our airlock system requires all data removed to be then fully anonymized via aggregation before leaving the NHS. All data used in research projects within the SDE are managed under our database research ethics committee approval and research office and data protection approvals. All access to data for research is approved by the National Institute for Health and Care Research Imperial Biomedical Research Centre data access committee chaired by the ICHT Caldicott guardian. All data access for validation and curation is approved by the Trust data protection office, Caldicott guardian, and transformation chief clinical information officer. Participants of the health care professional survey were required to provide informed consent before submitting the survey. Participants did not receive compensation or incentives to take part, responses were anonymized, and no personal or identifiable data were collected.

### Patient and Public Involvement, Engagement, and Participation

A patient and carer reference group was established to incorporate the views of women with lived experience of gynecological cancer; the group met quarterly during the 18 months of the project. From the outset, the patient and public involvement, engagement, and participation group was engaged in the project, beginning with the initial concept phase. Early on, they highlighted the challenges of fragmented clinical data records as a significant barrier to effective care. The women expressed support for data from medical records to provide a trusted source of accurate, up-to-date information in the ovarian cancer informatics platform. They appreciated the potential of the platform to save clinicians’ time during health care delivery and expressed strong support for consolidating information into a single, unified platform. They were supportive of deidentified data being used for audit and research. Throughout the project, we held 2 additional meetings with the group. During these sessions, we presented the proposed platform, actively seeking their feedback and suggestions. Their input was carefully considered and incorporated into the final design, ensuring that the platform addressed their concerns and needs. Specifically, the group highlighted the importance of referral information and visualization of genetic results, and this was incorporated into the final design.

## Results

### National Survey

#### Respondent Characteristics

Responses were received from 92 professionals in gynecological cancer (Table 1). As the link to the survey was distributed through the mailing lists of British Gynaecological Cancer Society, Institute of Cancer Research, Association of Cancer Physicians, The British Association of Gynaecological Pathologists, UK Oncology Nursing Society, British Society of Urogenital Radiology, and via social media (“X”), and it was not possible to ascertain how many professionals had the opportunity to take part but did not, a response rate could not be calculated. Of 92 respondents, 57.6% (53/92) were oncologists, 17.4% (16/92) were nurses, and 10.9% (10/92) were radiologists; the remaining 13 respondents had other diverse clinical roles. Half (46/92, 50.0%) had 11 years of experience or more in gynecological oncology and 73.9% (65/88; missing data: n=4) had more than 6 years of experience using EHRs. Table 1 illustrates the professional group and years of experience retrieving information from EHRs for clinical decision-making.

**Table 1.** Respondent characteristics.

Characteristics	Values, n (%)
Professional role	
Gynecological oncologist	26 (28.3)
Medical oncologist	19 (20.7)
Clinical oncologist	7 (7.6)
Consultant gynecologist	1 (1.1)
Obstetrics and gynecological trainee doing gynecological oncology ATSM^a^	2 (2.2)
Clinical nurse specialist	11 (12.0)
Gynecological oncology nurse	5 (5.4)
Radiologist	10 (10.9)
Research delivery	4 (4.3)
Dietitian	2 (2.2)
Pathologist	2 (2.2)
MDT^b^ coordinator	1 (1.1)
Oncology pharmacist	1 (1.1)
Palliative care physician	1 (1.1)
Missing data	0
Years of experience in gynecological cancer	
0‐2	16 (17.4)
3‐5	17 (18.5)
6‐10	13 (14.1)
11‐20	26 (28.3)
≥21	20 (21.7)
Missing data	0
Years of experience using electronic health records	
<1	5 (5.7)
1‐5	18 (20.5)
6‐10	29 (33.0)
>10	36 (40.9)
Missing data	4

a ATSM: advanced training skills module.

b MDT: multidisciplinary team.

Considering the day-to-day management of individual patients, 92.3% (84/91; missing data: n=1) of respondents said that they routinely access multiple electronic systems; 28.6% (26/91) reported using ≥5 systems. Around half (47/92, 51.1%) spend between 11% and 30% of their clinical time looking for information in electronic systems; however, 17.4% (16/92) said that they spend more than 50% of their clinical time looking for information in the EHR (Table 2). Table 2 highlights the number of systems accessed and the time spent looking for information in electronic systems.

**Figure 1.**
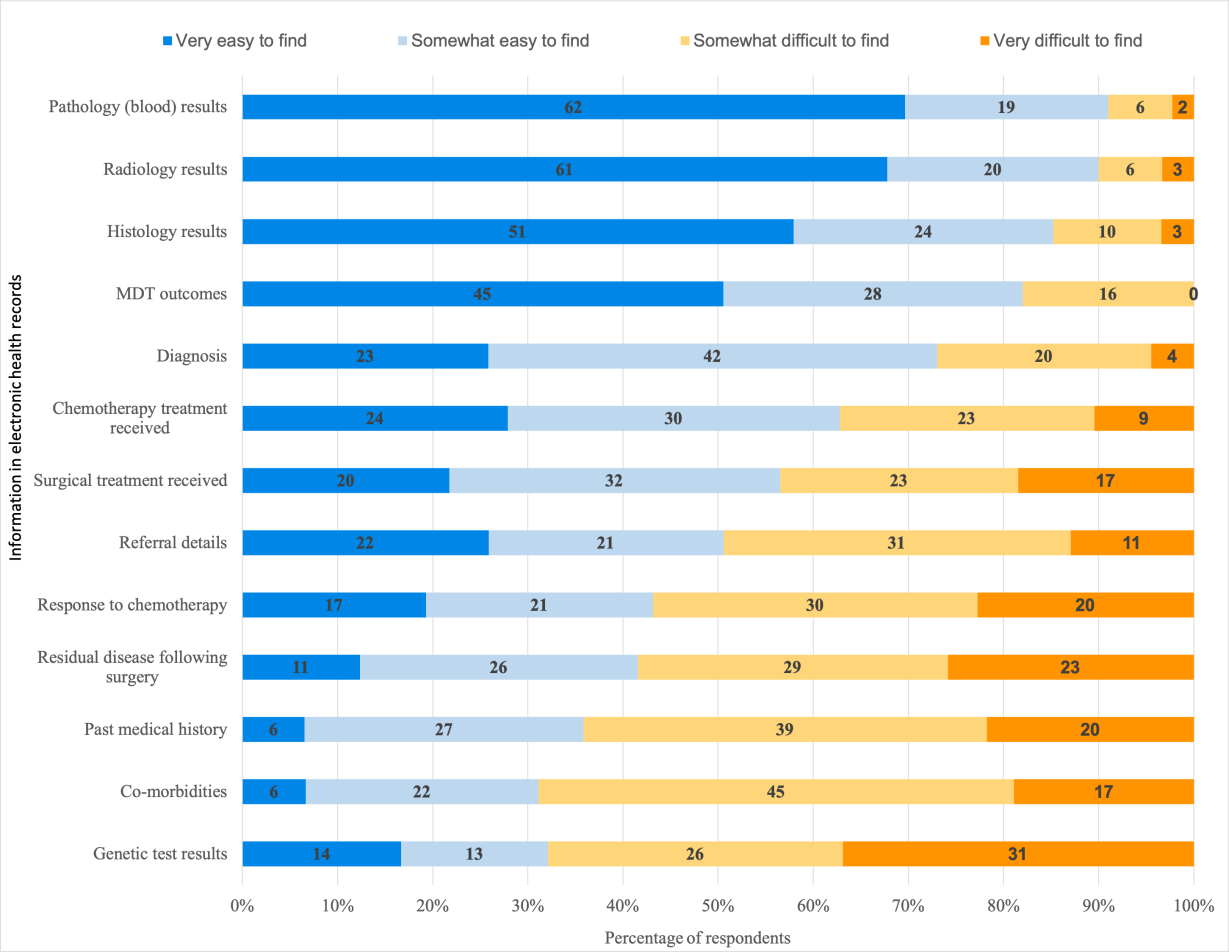
Finding different types of information in electronic health records: respondents were asked to consider their experience of searching for different components of patient information within electronic health record systems. MDT: multidisciplinary team.

**Figure 2.**
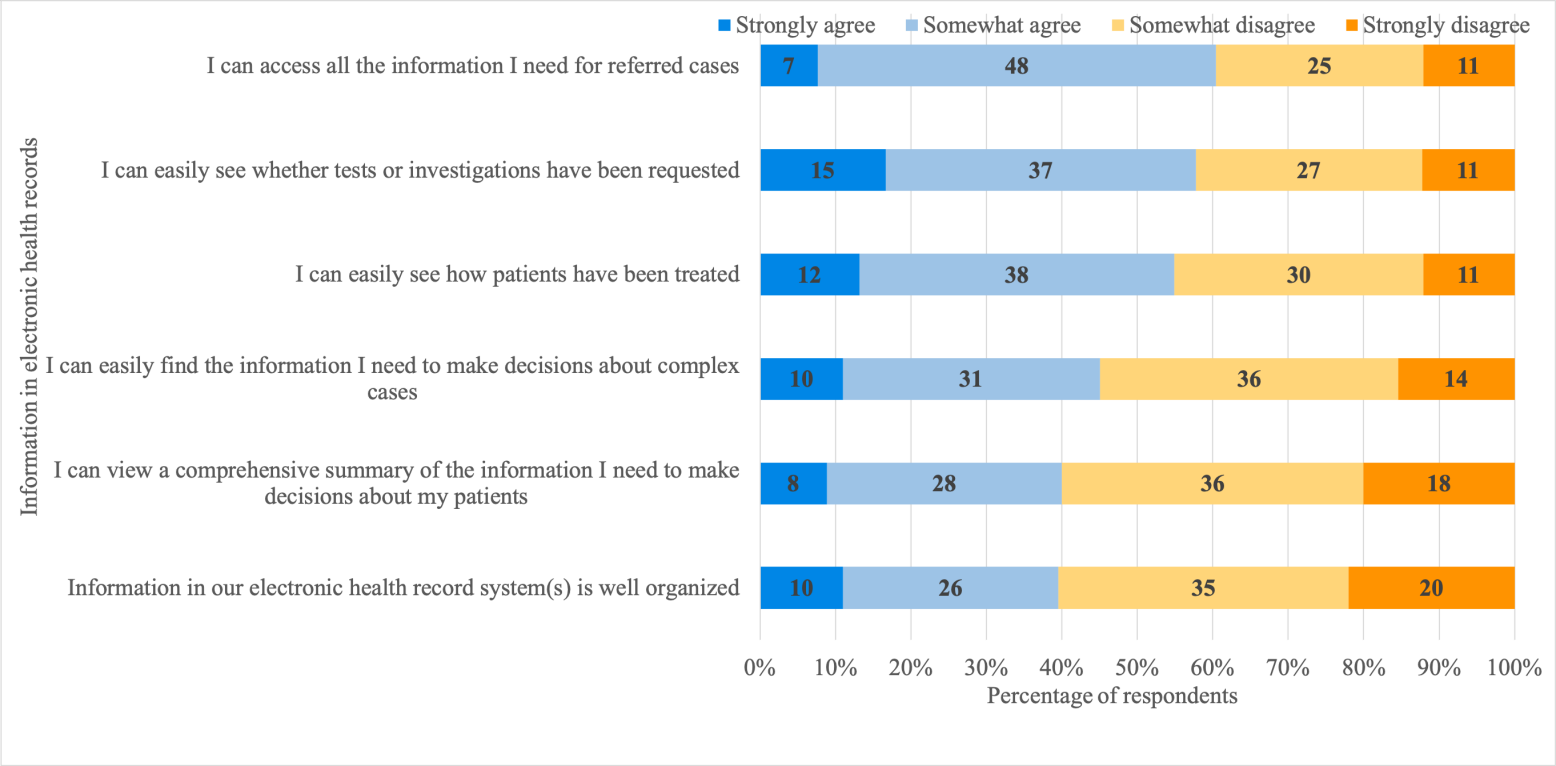
Experiences of using electronic health records for clinical decision-making: respondents were asked how easily they could access information in electronic health records.

When asked to consider how easily they can find different types of EHR information, more than 90% of professionals said that pathology (blood) and radiology results were easy to find, whereas patients’ past medical histories and comorbidities were reported by many (64.1% and 68.9%, respectively) as difficult to locate (Figure 1). Of the respondents who routinely look for genetic test results, 67.1% (57/85) said that finding them is difficult; 36.5% (31/85) said that genetic test results were *very* difficult to find. Only 10 respondents strongly agreed that information in their EHR systems is well organized and 60.0% (54/90; missing data: n=2) said that they do not have access to a comprehensive summary of individual patient information needed to make treatment decisions (Figure 2).

**Table 2.** Overview of electronic health record use.

Overview	Values, n (%)
How many electronic systems do you routinely access for patient management and clinical decision-making?	
1	7 (7.7)
2	15 (16.5)
3	22 (24.2)
4	21 (23.1)
≥5	26 (28.6)
Missing data	1
What proportion of your clinical time do you spend looking for information in electronic systems?	
0%‐10%	3 (3.3)
11%‐20%	25 (27.2)
21%‐30%	22 (23.9)
31%‐40%	14 (15.2)
41%‐50%	12 (13.0)
>50%	16 (17.4)
Missing data	0

### Challenges With Using EHRs

Respondents were asked for their views on the greatest challenges with using EHRs; 79.3% (73/92) answered this question. Interrater agreement for the qualitative coding of participants’ free-text responses was excellent (κ=0.97; *P*<.001). Across 73 responses, 141 words or sentence fragments related to challenges with EHR use; these sentence fragments were organized into 9 descriptive categories (Table 3).

Similar challenges were reported across the different health care professional roles. One-quarter of reported challenges (36/141, 25.5%) related to EHR use being time-consuming or difficult, particularly in relation to accessing or finding information and having to use multiple different systems:

*The time required to find all the info—only have secs to mins [sic.] for each patient.*

[Respondent 56, radiologist]

*Most [systems] are complex and each site is different. It takes a lot of time to get used to each system.* 

[Respondent 21, radiologist]

A further 25% (35/141) described challenges relating to using multiple EHR systems, the most frequently reported challenge being lack of system interoperability:

*Far too many systems that don’t speak’ with each other*.

[Respondent 46, clinical nurse specialist]

A few reports (8/141, 5.7%) related to the negative impacts of EHR use on the quality of care, for example, delays in care delivery (3 mentions) and the increased risk of error (2 mentions). One oncologist’s response reflects a concise summary of the key challenges with EHR use that emerged from the survey, namely, lack of interoperability, data fragmentation, and reduced efficiency:

*lack of interoperability and fragmentation of the patient record across multiple systems leading to increased time required to put together a clinical picture and reduced effectiveness and productivity in clinic as a result.*

[Respondent 61, gynecological oncologist]

**Table 3.** Challenges with electronic health records: summative content analysis.

Descriptive category	Reported challenge	Count (%) of reported challenges (n=141)
Time-consuming or difficult to:	Access or find information	16 (11.3)
	Use or get used to multiple systems	10 (7.1)
	Retrieve information for patients treated at multiple institutions	5 (3.5)
	Put together a clinical picture	2 (1.4)
	Time-consuming (general comment)	3 (2.1)
	Category total	36/141 (25.5)
Multiple EHR^a^ systems	Lack of interoperability	27 (19.1)
	Manual entry of information from other systems	4 (2.8)
	Multiple logins or passwords to access information	4 (2.8)
	Category total	35/141 (24.8)
Problems with ICT^b^ infrastructure	Hardware issues and computer availability	6 (4.3)
	System crashes	4 (2.8)
	Internet issues	4 (2.8)
	Software upgrades	4 (2.8)
	Slow speed of systems	2 (1.4)
	Slow IT support when locked out	1 (0.7)
	Category total	21/141 (14.9)
Poor usability	Interface is not easy to use	7 (5.0)
	Information poorly organized	3 (2.1)
	Complex system	1 (0.7)
	Workarounds are required	1 (0.7)
	Category total	12/141 (8.5)
Poor data quality	Inaccurate	3 (2.1)
	Out-of-date	5 (3.5)
	Missing	1 (0.7)
	Duplication	1 (0.7)
	Incorrect diagnostic codes	1 (0.7)
	Category total	11/141 (7.8)
Lacking system features	No system that does everything	3 (2.1)
	No timelines or overview of patient journey	2 (1.4)
	No patient summaries	2 (1.4)
	Important information not highlighted	1 (0.7)
	Category total	8/141 (5.7)
How staff use the system	Poor documentation quality	3 (2.1)
	Documentation in the wrong place	1 (0.7)
	Variation in documentation practices	1 (0.7)
	Variation in coding practices	1 (0.7)
	System not used as intended	1 (0.7)
	Different levels of access	1 (0.7)
	Category total	8/141 (5.7)
Impact on quality of care	Increases risk of error	2 (1.4)
	Delays in care delivery	3 (2.1)
	Limits number of patients that can be seen in clinic	1 (0.7)
	Consultations are less effective	1 (0.7)
	Negatively affects rapport with patient	1 (0.7)
	Category total	8/141 (5.7)
Digital transformation	Incomplete EHR adoption	1 (0.7)
	Governance and data sharing	1 (0.7)
	Category total	2/141 (1.4)

a EHR: electronic health record.

b ICT: information and communication technology.

### Benefits of EHRs

Respondents were asked to consider the greatest benefits with using EHRs; 79.3% (73/92) answered this question. Interrater agreement for response coding was excellent (κ=0.91; *P*<.001). From 73 responses, 117 words and sentence fragments described the benefits of EHR use; these benefits are organized into 7 descriptive categories (Table 4). Six descriptive categories represent *actual* benefits reported by respondents; the seventh descriptive category represents responses that were caveated in some way—that is, the *potential* benefits of EHRs. The most frequently reported benefit was *ease of access to patient information* (55/117, 47.0%) while 18.8% (22/117) of respondents reported that EHRs are *better than paper notes*. Around 16% (19/117, 16.2%) of reported benefits were caveated, for example:

*in theory access to all you need but this is cumbersome at present*.

[Respondent 14, medical oncologist]

*theoretically should be able to produce audit data etc. but suspect there will be a large learning curve here*.

[Respondent 5, medical oncologist]

**Table 4.** Benefits of electronic health records: summative content analysis of free-text responses.

Descriptive category	Reported benefit	Count, n (%) of reported benefits (n=117)
Ease of access to information in the patient record	Ease of access	26 (22.2)
	Information is readily available	10 (8.5)
	Information is in one place	10 (8.5)
	Information can be easily shared	3 (2.6)
	Multiple people can access a patient record at the same time	2 (1.7)
	Information can be easily organized	2 (1.7)
	Patient access	1 (0.9)
	Can easily check historical records	1 (0.9)
	Category total	55/117 (47.0)
Better than paper notes	Easier than paper notes	8 (6.8)
	Legible	6 (5.1)
	Cannot get lost	5 (4.3)
	Do not need to request notes	3 (2.6)
	Category total	22/117 (18.8)
Benefits for clinical staff	Time-saving	8 (6.8)
	Support clinical decision-making	1 (0.9)
	Category total	9/117 (7.7)
Secondary use of EHR^a^ data	Research, audit, service evaluation, and quality improvement	6 (5.1)
	Category total	6/117 (5.1)
Improved care quality	Improved care quality	3 (3.6)
	Category total	3/117 (2.6)
Other benefits	Data security	1 (0.9)
	Real-time information	1 (0.9)
	Structured data	1 (0.9)
	Category total	3 (2.6)
Potential or caveated benefits	Useful if you can access or find the information needed	5 (4.3)
	Potential use for research, audit, and evaluation	5 (4.3)
	Potential to be time-saving	2 (1.7)
	Easy once you are used to it	2 (1.7)
	Benefits outweigh the challenges	2 (1.7)
	Interface could be more user-friendly	1 (0.9)
	Potential for safer care delivery	1 (0.9)
	Information could all be in one place	1 (09)
	Category total	19/117 (16.2)

a EHR: electronic health record.

### Risks Associated With EHRs 

Respondents were asked to comment on any perceived risks with using EHRs; 65.2% (60/92) answered this question giving rise to 81 words and sentence fragments about EHR risks. Interrater agreement for response coding was excellent (κ=0.92; *P*<.001). Seven descriptive categories were used to summarize the data (Table 5). Nearly a third of reported risks (24/81, 29.6%) related to poor EHR data quality and the inherent risks to patient safety from incorrect, missing, or out-of-date documentation:

*Everyone copy and pastes information that isn’t always up to date and accurate*.

[Respondent 32, dietician]

*inaccurate or out of date information. This can lead to errors in care and I have seen this personally with incorrect info added to a patient’s record that affected care*.

[Respondent 37, gynecological oncologist]

Around 20% of reported risks related to data security (17/81, 21.0%): “eSecurity, risk of hacking etc*”* (Respondent 60, gynecological oncologist), or system failure (14/81, 17.3%): “When systems are down there is no way of knowing what is happening to the patient” (Respondent 29, clinical oncologist). Six respondents (6/60, 10%) perceived there to be no risks with using EHRs.

Overall, the findings of the national survey aligned with our local gynecological oncology team’s experiences with the challenges, benefits, and risks of EHRs. These findings suggest that current EHR systems may not be optimally designed to present critical clinical information in an easily accessible manner. The survey highlighted the need to improve data integration, enhance user interface, standardized data entry, and improve interoperability.

**Table 5.** Risks of electronic health records: summative content analysis of free-text responses.

Descriptive category	Reported risk	Count, n (%) of reported risks (n=81)
Poor quality data	Incorrect data entry—including copy and paste errors	13 (16.0)
	Information missing or out-of-date	11 (13.6)
	Category total	24/81 (29.6)
Data security	Data security	17 (21.0)
	Category total	17/81 (21.0)
System failure	Data security	14 (17.3)
	Category total	14/81 (17.3)
Negative impacts for clinical staff	Risk of overlooking important information	5 (6.2)
	Detrimental to mental health	2 (2.5)
	Reduced productivity	2 (2.5)
	Category total	9/81 (11.1)
Negative impacts for delivery of care	Too many clinical systems create safety risk	3 (3.7)
	Errors or omissions affecting care	3 (3.7)
	Patients are not reviewed in person	1 (1.2)
	Category total	7/81 (8.6)
Other risks	Public mistrust about how data are used	2 (2.5)
	Financial impacts of poorly functioning EHR^a^	1 (1.2)
	Staff recruitment and retention is negatively affected	1 (1.2)
	Category total	4/81 (4.9)
No risks	No risks	6 (7.4)
	Category total	6/81 (7.4)

a EHR: electronic health record.

### Ovarian Cancer Platform Prototype

The survey highlighted a lack of a comprehensive patient summary in existing EHR and difficulty in finding clinical information necessary for patient care; for example, 67% (57/85) of respondents found locating genetic test results difficult, but these results are required for clinical care. These challenges stem from poor interoperability stemming from fragmented data silos, inconsistent terminologies, unstructured clinical notes, and manual workflows that burden clinicians. We sought to address the problem of data fragmentation and lack of interoperability of multiple EHRs by developing an integrated informatics platform to support clinical decision-making in ovarian cancer.

The clinical stakeholders identified key factors to be included within a single platform for clinical decision-making for a patient with ovarian cancer. Next, the informatics team identified the location of the relevant data within the different source clinical systems. Both structured and unstructured (free text) data were explored and curated within the iCARE SDE. Rule-based algorithms and NLP were used to transform free-text unstructured data such as genetic data and operation notes. The data points, type of data, and their location within the clinical systems are summarized in Table 6. Table 6 displays the types of data required for clinical decision-making, their location within the clinical records, and how the data should be visualized within the platform.

**Figure 3.**
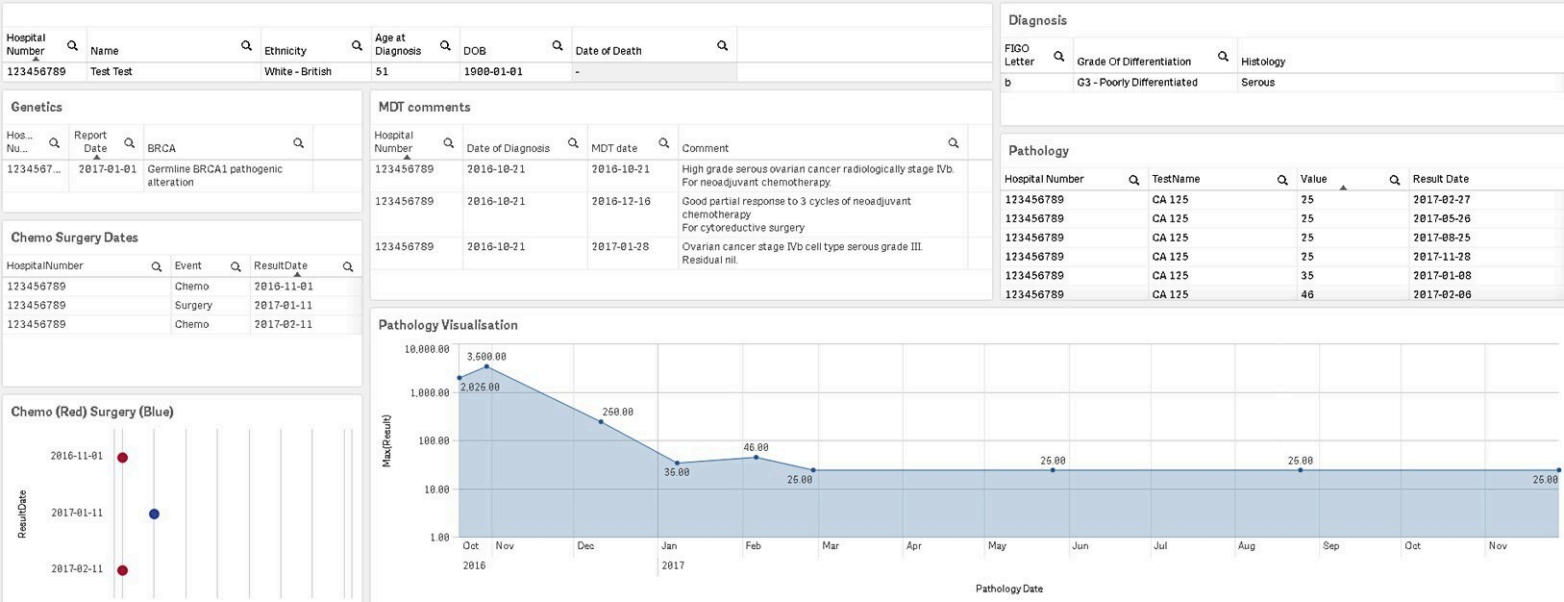
Ovarian cancer informatics platform prototype—individual patient summary view (synthetic data).

Draft sketches of a proposed platform were created by the clinical team using synthetic data (Multimedia Appendix 2). Visualization of the data was developed by the data engineers based on clinician feedback. The final platform was developed through an iterative process and brings together important documentation from various clinical systems and presents it back to clinicians in a single location (Figure 3). The individual patient summary view displays key information for a patient with ovarian cancer including patient demographic details, cancer diagnosis, genetics, pathology results, MDT comments and decisions, and treatment (chemotherapy and surgery). This proposed platform will be further developed and integrated in line with ICHT standard operating policies. Usability, utility, and risk assessment of this platform will be evaluated by the direct care team prior to implementation into clinical practice.

**Table 6.** Development of an informatics platform for decision-making for patients with ovarian cancer.

Type of data	Data points	EHR^a^ system	Type of data	Clinical team request for visualization of data
Diagnostic data	Date of diagnosis	MDT^b^ reporting system (Somerset Cancer Register)	Structured	Single field displaying data element
	Histology	MDT reporting system (Somerset Cancer Register)	Structured	Single field displaying data element
	FIGO^c^ stage	MDT reporting system (Somerset Cancer Register)	Structured	Single field displaying data element
Demographics	Age	EHR (Cerner Millenium)	Structured	Single field displaying data element
	Ethnicity	MDT reporting system (Somerset Cancer Register)	Structured	Single field displaying data element
Outcome	Date of death	NHS Spine	Structured	Single field displaying data element
Treatment	Sequencing of treatment	Chemotherapy management prescribing system (Aria oncology) EHR (Cerner Millenium)	Unstructured	Graphic to represent treatment sequence
	SACT^d^	Chemotherapy management prescribing system (Aria oncology)	Structured data	Link for further information: Name of SACT Date administered Number of cycles Dose
	Surgery	EHR (Cerner Millenium)	Structure and unstructured data	Type of surgery and date of surgery Allow for multiple entries Click a link for operation notes
	MDT discussions	MDT reporting system (Somerset Cancer Register)	Unstructured	Option of multiple MDT summaries with dates
Genetics	Germline and tumor genomics (including *BRCA^e^* results)	EHR (Cerner Millenium)	Unstructured	Gene tested Result to be grouped: Pathogenic alteration No pathogenic alteration Variant of unknown significance
Investigations	Blood results Hematology Biochemistry Tumor markers	Pathology systems (North West London pathology systems)	Structured	Value + date Option to graph data to look for trends over time

a EHR: electronic health records.

b MDT: multidisciplinary team.

c FIGO: International Federation of Gynecology and Obstetrics.

d SACT: systemic anticancer therapy.

e BRCA BReast CAncer gene.

## Discussion

### Summary of Key Findings

This study explored multiprofessional perspectives on using EHRs in gynecological oncology; an informatics platform was co-designed to support clinical decision-making in ovarian cancer. Our survey indicates that most gynecological oncology professionals use multiple EHR systems and spend a significant amount of time locating information in different clinical systems. Key challenges were interoperability and fragmentation of the patient record across disparate systems—meaning retrieval of pertinent information for clinical decision-making can be time-consuming. Some expressed concerns about the challenges, delays in care delivery, and the risk to patient safety. The main benefit of EHR use was perceived to be ease of access; however, most respondents did not have access to a comprehensive patient summary to support clinical decision-making. While many EHR issues highlighted here are already well recognized, this is the first study to explore EHR use in gynecological oncology, and the novelty of our work lies in the co-design of an integrated ovarian cancer informatics platform to address these problems.

### Comparison With the Wider Literature

Consistent with existing literature, our survey of UK gynecological cancer professionals identifies several challenges associated with EHR use, namely, the need to access and use multiple EHR systems, lack of system interoperability, and difficulty finding pertinent information for clinical decision-making [26-28]. Cancer care involves MDTs and patient interactions across a variety of departments (imaging, surgery, chemotherapy, etc) and health care settings; however, a single EHR system rarely supports documentation across the entire patient pathway [28]. Oncologists in the United States also report that contemporary EHRs fail to support care coordination between health care organizations with significant implications for patient management since one organization’s input may influence another’s treatment recommendation [8,14]. Some have argued that interoperability issues may have a particularly detrimental impact on patients with cancer due to the volume and complexity of oncology data; patients with cancer often feel compelled to maintain a record of all their medical information to be able to access vital cancer care [13]. In the United Kingdom, NHS policy and technology advancements over the last 20 years have resulted in a complicated EHR ecosystem, with multiple vendors, ancillary systems, and third-party applications being used in oncology practice. Open EHR standards such as Fast Health Interoperability Resources, which organize data elements so that they are easily understood by recipient systems, are contributing to EHR integration, but progress is invariably slow [7,29].

In our survey, UK-based oncology professionals reported similar challenges as colleagues in the United States regarding information overload and difficulties locating pertinent EHR data [14]. More than a quarter of gynecology health care professionals report using 5 or more different systems. We did not collect the specific details regarding the EHRs used; however, as there is no standard national EHR system in the United Kingdom, these systems are likely to vary in their complexity. It is cognitively challenging and time-consuming for oncology professionals to find and synthesize multiple data EHR sources (pathology, radiology, genomics, and clinical notes) that are critical for decision-making [30]. Many oncology professionals are concerned about overlooking relevant information due to the volume of data and multiplicity of EHR systems [14,30]. Furthermore, a single piece of information can be entered in multiple locations in the patient record, raising questions about the “ground truth” where data are not congruent and complicating efficient retrieval [31]. Effective cancer treatment and management require exchange of large amounts of information in a timely manner; however, cancer data are generally not standardized and unstructured data are “buried” in clinical notes, images, and laboratory reports [14]. In some cases, EHRs contain relevant information for clinical decision-making, but these data remain “hidden” to treating professionals; for example, according to a retrospective case note review of 299 patient records, half of the women whose EHR record contained dispersed risk factor information meeting the criteria for genetic evaluation for heritable forms of breast and ovarian cancer were not referred for testing [27]. As our survey has highlighted, where genetic testing has been undertaken, there is often difficulty locating test results in the patient record, and this can be explained by the lack of interoperability between systems in the Genomic Laboratories and NHS trust and the current requirement to manually upload results—risking treatment delays. These examples demonstrate the need for critical EHR information to be prominently and clearly displayed, unobstructed by less important data [14,27].

### Recommendations and Future Work

There is broad acceptance that EHR implementation is not solely a technical undertaking and that clinical systems must be adapted to meet specific end user needs regarding efficiency and ease of use; however, while progress in this area remains slow, clinical teams will continue to develop workarounds so that health care professionals have the information they need for clinical decision-making and care delivery [32]. Our informatics platform for ovarian cancer aims to address known challenges relating to data fragmentation and retrievability, informed by oncology professionals’ data requirements for clinical decision-making. ICHT has established interoperable data platforms that will enable this work. This project is at a preliminary stage and focused on developing an informatics platform in response to a clinical need. Implementation considerations extend beyond the intended scope of this manuscript. The next steps for the planned work are to implement the platform into clinical practice and evaluate its clinical impact and workflow improvement. We will address emerging problems during the agile implementation process as well as consider challenges with less digitally mature institutions. The data feeds are taken from systems used in routine care and therefore deemed accurate as part of the patient’s EHR. Pipelines were validated by the direct care team, who tested before and after samples of data to ensure accuracy. If discrepancies were observed, these were passed to the NHS data engineering team who then tested and corrected issues. Pipelines of validated data were automated, ensuring replicability with error alerting built in. We recognize that pipelines will need to be periodically checked and validated in line with assessments made in the future clinical safety review once the platform is operational.

### Evaluation Plans

We will perform a formal evaluation of the proposed platform, which will include risk assessment, formative usability testing, and summative controlled evaluation.

#### Risk Assessment

This will include clinical validation for all the data fields. Ovarian cancer clinicians will independently benchmark the data held within the informatics platform against EHR. For example, 3 independent ovarian cancer clinicians, blinded to each other, will be given 40 randomly selected patients’ records that represent diverse cancer stages and treatment history. The clinicians will extract and populate structured key data points from both the platform and the EHR. Accuracy, completeness, and timeliness metrics will be evaluated in platform versus EHR.

#### Formative Usability Testing

Assessment will include the systems usability scale questionnaire and think-aloud interviews with follow-up questions. For example, 10‐15 representative end users that include oncologists, surgeons, nurses, and MDT coordinators will be tasked with realistic clinical scenarios. Sekhon’s theoretical framework will be used to guide the topics and thematic analysis of the transcript.

#### Summative Controlled Evaluation

This will measure the effect of the introduction of the informatics platform on distribution of clinic and MDT preparation activities (searching for information and formulating treatment plans). We will perform a pretest and posttest time monitor study. Time-motion data will be collected by an external observer to record the time per patient in preparing for clinic and MDT discussions. Multilevel regression will be used to examine time and activity distribution before and after the implementation of the informatics platform to assess the benefits in terms of time-saving and reducing clinician burden.

Once evaluated, we plan to implement the informatics dashboard as part of MDT and clinic preparation. Structured quality improvement metrics with defined process (adoption rates), outcome (time to prepare), and balancing (monitoring for unintended consequences) measures will be embedded to provide ongoing feedback. The principles behind the design approach are transferable to other tumor types. Although many of the data fields might be consistent, a human-centered approach will ensure that the development of other platforms contains all critically relevant information. To mitigate against digital tools contributing to stress and burnout, there must be careful examination of usability, impact on clinical workflow, and effectiveness in terms of time-savings and productivity; these are areas of focus for our future work [33].

To enhance clinical decision-making for individual patients and to generate real-world evidence to drive improved outcomes in oncology, the next generation of EHR systems should embed co-designed CDSSs with functionality that synthesizes fragmented EHR data, presenting them to clinicians for review and action. Redesign of existing EHRs will also need to integrate genomics data and synthesize these data with clinical documentation to support an era of personalized care guided by genetic testing [3]. Since most EHR data are unstructured, specialized data extraction tools will be necessary to derive meaningful information. NLP tools can extract information from free-text clinical narratives and have the potential to save health care professionals' valuable time [1]. Analogous to streamlining decision support in aviation, Shulman explains, “in the cockpit design of modern passenger jets, all instruments are readily accessible but only the instruments showing the most critical data are directly in front of the pilots*….*” [14]. Until such time that reengineered, context-based EHRs can use biomedical ontologies and disease models to determine the most relevant parts of the record to display, there will be a demand for decision support tools such as ours to present essential data for decision-making in a single location.

### Strengths and Limitations

This is the first study to report gynecological oncology professionals’ experiences of EHR use, and based on these findings, we have co-designed an integrated informatics platform to drive improvements in quality and outcomes related to the most lethal gynecological malignancy in the United Kingdom. We used a survey design to increase understanding of EHR use; while survey designs can risk lacking detail on the topic being investigated, our findings are demonstrably consistent with in-depth qualitative studies using interviews to explore the same topic [8,9,34,35]. The survey was administered on the web via social media and mailing lists of relevant gatekeepers’ limitations therefore exist. First, it was not possible to calculate a response rate, and while a larger sample size could have enhanced reliability and subtheme analysis, we used a mixed methods approach involving diverse professionals, and saturation was deemed satisfactory. Second, self-reported data can be influenced by subjective perceptions, discrepancies with EHR records, and informed presence bias. Third, bias may have been introduced through variation in the interpretation of “using” an EHR, which can encompass accessing an EHR to view test results with paper notes for clinical documentation through to the use of an EHR with comprehensive functionality. Studies show conflicting findings on EHR efficiency, with some reporting reduced documentation time and others perceiving increased workload. To address these biases, researchers recommend combining self-reported data with objective EHR usage metrics, conducting time-motion studies, and applying bias adjustment methods to improve accuracy and reliability of findings [36,37].

In addition, generalizability is difficult to assess since the sample size was small and the geographical location of respondents was not collected; however, the findings are representative of diverse professional groups across medicine, radiology, nursing, and other disciplines. Although this study was undertaken in the NHS setting in the United Kingdom, health care systems around the world are facing the same issues of interoperability, data fragmentation, and laborious information retrieval. The human-centered design principles used in this study to co-design our integrated informatics platform for ovarian cancer could be used in other clinical specialties to develop specific platforms to drive improvement in quality and outcomes.

### Conclusions

To make informed decisions about the management of patients, clinicians need the right information, in the right place, at the right time. EHR systems are not currently optimized to support timely and effective decision-making in gynecological oncology. Co-designed clinical decision support tools could address challenges with data fragmentation and information retrieval to reduce administrative inefficiencies and increase satisfaction with health information technology among oncology professionals.

## Supplementary material

10.2196/58657 Multimedia Appendix 1 Survey questionnaire.

10.2196/58657 Multimedia Appendix 2 Draft sketches of the informatics platform created by the clinical team.

## Abbreviations:

CDSS: clinical decision support system
EHR: electronic health record
iCARE: Imperial Clinical Analytics Research and Evaluation
ICHT: Imperial College Healthcare NHS Trust
MDT: multidisciplinary team
NHS: National Health Service
NLP: Natural language processing
SDE: secure data environment
