# Supplementary material for: Understanding and Addressing Challenges With Electronic Health Record Use in Gynecological Oncology: Cross-Sectional Survey of Multidisciplinary Professionals in the United Kingdom and Co-Design of an Integrated Informatics Platform to Support Clinical Decision-Making
Source: JMIR Cancer. 2025 Sep 10;11:e58657. doi: 10.2196/58657 (PMC12422591; doi:10.2196/58657)
Supplement: Multimedia Appendix 1 [file cancer-v11-e58657-s001.pdf]

Imperial College London

Your Opinion Matters: Using Electronic Health Records in the Management of Ovarian Cancer

Clinical Lead: Dr Laura Tookman | Chief Investigator: Dr Rachael Lear
Sponsor reference:
Version 6 | 04.04.2023

To make informed and safe decisions, clinicians need the right information about the right patient in the right place at the right time.

A team of clinicians, researchers, and data scientists at Imperial College London are exploring the use of electronic health records in the management of patients with ovarian cancer.

If you are a professional involved in treating patients with ovarian cancer, we would like to understand your perspectives and experiences.

We will use the information you provide in developing strategies to bring together the most important routine data for clinical decision-making in ovarian cancer.

This questionnaire takes around 7 minutes to complete and all responses are anonymous. The full study information sheet can be downloaded here: [insert link]

Thank you for your time and support in completing this survey.

By completing this questionnaire, you agree for us to use the information you provide in our analyses, publications and presentations relating to this project. Please note that as the questionnaire is anonymous, you will not be able to withdraw your responses from the study at a later date. [tick box]

I agree – proceed to question 1
I disagree

Q1:
Please select your current position [tick box]

Medical oncologist
Clinical oncologist
Gynae oncologist
Clinical nurse specialist
Dietician
Physiotherapist
Pathologist
Radiologist
MDT coordinator
Other [free-text] ……….

Q2:
Years of experience in gynaecological cancer [tick box]
0-2 years
2-5 years
6-10 years
11-20 years
>20 years
I do not work in this field

Q3:
Number of years using electronic health record systems [tick box]
<1 year
1-5 years
6-10 years
>10 years

The first few questions address your experiences of using electronic health record systems for the management of ***individual patients with a confirmed diagnosis of ovarian cancer.***

Q4:
How many electronic systems do you routinely access for patient management and clinical decision-making? (e.g. radiology, pathology, prescribing, etc)
[tick box]

1
2
3
4
5 or more

Q5: What proportion of your clinical time would you say you spend looking for information in electronic systems?
[tick box]

0-10%
10-20%
20-30%
30-40%
40-50%
More than 50%

Q6
Considering the electronic health record systems that you currently use, what are your experiences of searching for the following patient information?
[tick box]

	Very difficult to find	Somewhat difficult to find	Somewhat easy to find	Very easy to find	I do not routinely look for

					this information
Referral details					
Diagnosis					
Co-morbidities					
Past medical history					
Pathology (blood) results					
Histology results					
Radiology results					
Genetic test results					
Surgical treatment received					
Residual disease following surgery					
Chemotherapy treatment received					
Response to chemotherapy					
MDT outcomes					

Q7
Considering the electronic health record systems that you currently use, to what extent do you agree with the following:
[tick box]

	Strongly agree	Somewhat agree	Somewhat disagree	Strongly disagree
I can access all the information I need for referred cases				
I can easily see whether tests or investigations have been requested				
I can easily see how patients have been treated				
I have to access multiple systems to obtain information for the management of individual patients				
Information in our electronic health record system(s) is well organised				
I can easily find the information I need to make decisions about complex cases				
Our electronic health record system(s) make it easy for the MDT to discuss complex cases.				
I can view a comprehensive summary of the information I need to make decisions about my patients				
A comprehensive patient summary would be useful for day-to-day decision-making.				
A comprehensive patient summary would be useful for MDT meetings.				

Q8
If you were able to view a patient summary containing key information to help with clinical decision-making, which information would you like to see displayed?

	Not important to display	Somewhat important to display	Very important to display	Essential to display
Stage at diagnosis				
Pathology				
Baseline tumour markers				
Radiology findings				
Performance status				
Initial treatment: surgery/chemotherapy				
Whether surgery was performed				
Residual disease following surgery				
Other operation details				
Trend in tumour markers				
Trends in other bloods (e.g. albumin, Hb, etc)				
Current chemotherapy treatment				
Response to chemotherapy				
Genetic test results				
Co-morbidities				
Details of clinical trial enrolment				

Q9
Is there any other information that you feel would be essential to include in a patient summary?
[free-text]

Q10
Is there anything else you would like to tell us about retrieving information from electronic health records for the management of individual patients?
[free-text]

The next questions are about collecting data for audit, service evaluation and research in ovarian cancer.

Q11
If it were possible to collect data automatically (i.e. not manually) for audit, service evaluation or research in ovarian cancer, which data would it be important to routinely collate?

	Not important	Somewhat important	Very important	Essential
Source of referral (e.g. GP, A&E)				
Patient age				
Patient ethnicity				
Co-morbidities				
Perormance status				
Risk factors				
Genetic test results				
Stage of cancer at presentation				
Cancer histology				
Lines of previous treatment				
Rates of primary/interval/secondary debulking surgery				
Rates of no surgery, no chemotherapy				
Rates of chemotherapy only				
Response to chemotherapy				
Surgical outcomes				
Trends in systemic anti-cancer treatments & outcomes				
Details of clinical trial enrolment				
30-day mortality				
Survival (progression-free survival, overall survival)				

Q12
Is there any other information that you feel would be useful to routinely collate for audit, service evaluation or research purposes?
[free-text]

Q13
Finally, is there anything else you would like to tell us about retrieving information from electronic health records for audit, service evaluation or research?
[free-text]

The last three questions ask you to consider electronic health records in the context of patient management and clinical decision-making:

Q14:
In your view, what are the **greatest challenges** (if any) with using electronic health records?
[free text]

Q15:
In your view, what are the **greatest benefits** (if any) with using electronic health records?
[free text]

Q16:

In your view, what are the **greatest risks** (if any) associated with using electronic health records?
[free text]

By clicking the ‘Submit’ button you provide your consent to participate in this survey.

Survey results will be circulated via the same mailing lists in the coming months.

If you are interested finding out more about this work, please contact < >
