# Supplementary material for: Understanding and Addressing Challenges With Electronic Health Record Use in Gynecological Oncology: Cross-Sectional Survey of Multidisciplinary Professionals in the United Kingdom and Co-Design of an Integrated Informatics Platform to Support Clinical Decision-Making
Source: JMIR Cancer. 2025 Sep 10;11:e58657. doi: 10.2196/58657 (PMC12422591; doi:10.2196/58657)

Dashboard ideas

Initial draft sketches created by the clinical team using synthetic data

2 potential aims

1. Patient specific details – To contain all the information that would be needed to manage the patient

2. Qlik Dashboard ideas:
   - to review service delivery
   - To identify inequalities
   - To answer clinically relevant questions
   - To aid audit/service improvement

Patient details (synthetic patient data)

Name: ………
DOB: XX/XX/XXXX
Identifier:
Gender: X
Ethnicity:
Germline status:…
Medication list (clickable link)
Comorbidity: ….

Tumour details

Date: XX/XX/XXXX
Histology: Serous Grade: high grade
Stage: XX
Baseline Ca125: XXX
First treatment: Laparotomy/ primary surgery
First chemotherapy: Carbo/taxol 3-weekly
Tumour genomics: XXXX

Treatment timeline

XX/XX/XXXX – e.g. laparotomy (there would be a clickable link here to the op notes)
XX/XX/XXXX – e.g. carboplatin + paclitaxel 3-weekly (clickable link which brings up number of cycles and dose modifications)
XX/XX/XXXX – Maintenance treatment

Imaging history

Date range: XX/XX/XXXX – XX/XX/XXXX (this would allow user to select range. Table would be scrollable to fit in window. Links to reports)

Date	Modality	Conclusion
XX/XX/XXXX	CT CAP	
XX/XX/XXXX	CT CAP	
XX/XX/XXXX	US liver	

Bloods

Date range: (this would allow user to select range)

Bloods 1: Ca125 (this would allow user to select bloods)

Bloods 2: Ca125 (this would allow user to select bloods)

Text: Treatment (this would allow user to select annotation text e.g. imaging, treatment

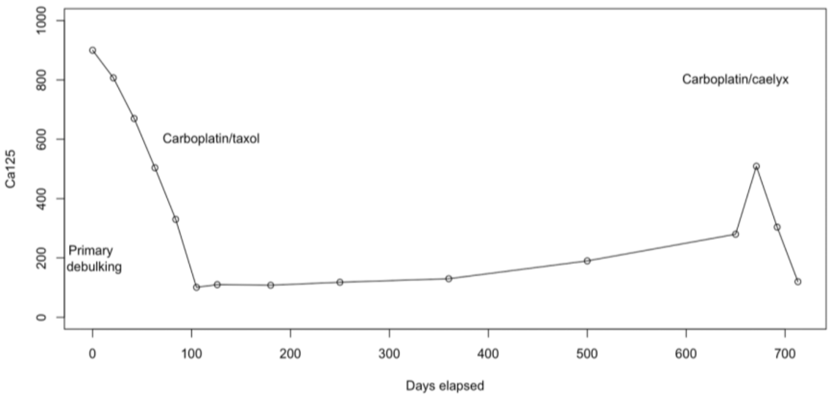

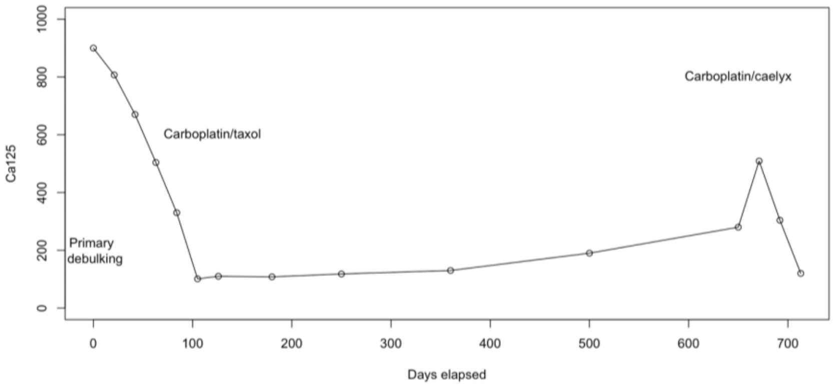

Supplement: Multimedia Appendix 2 [file cancer-v11-e58657-s002.pdf]
